# Silencing of a Cotton Actin-Binding Protein GhWLIM1C Decreases Resistance against *Verticillium dahliae* Infection

**DOI:** 10.3390/plants11141828

**Published:** 2022-07-12

**Authors:** Tingyan Cao, Minghui Qin, Shuai Zhu, Yuanbao Li

**Affiliations:** State Key Laboratory of Ecological Pest Control for Fujian and Taiwan Crops, Key Laboratory of Ministry of Education for Genetics, Breeding and Multiple Utilization of Crops, Plant Immunity Center, Fujian Agriculture and Forestry University, Fuzhou 350002, China; cty18855792177@163.com (T.C.); qinminghui657@163.com (M.Q.); shuaiz22620@126.com (S.Z.)

**Keywords:** cotton, *V. dahliae*, LIM protein, actin cytoskeleton, defense response

## Abstract

LIM proteins are widely spread in various types of plant cells and play diversely crucial cellular roles through actin cytoskeleton assembly and gene expression regulation. Till now, it has not been clear whether LIM proteins function in plant pathogen defense. In this study, we characterized a LIM protein, GhWLIM1C, in upland cotton (*Gossypium hirsutum*). We found that GhWLIM1C could bind and bundle the actin cytoskeleton, and it contains two LIM domains (LIM1 and LIM2). Both the two domains could bind directly to the actin filaments. Moreover, the LIM2 domain additionally bundles the actin cytoskeleton, indicating that it possesses a different biochemical activity than LIM1. The expression of *GhWLIM1C* responds to the infection of the cotton fungal pathogen *Verticillium dahliae* (*V. dahliae*). Silencing of *GhWLIM1C* decreased cotton resistance to *V. dahliae*. These may be associated with the down regulated plant defense response, including the *PR* genes expression and ROS accumulation in the infected cotton plants. In all, these results provide new evidence that a plant LIM protein functions in plant pathogen resistance and the assembly of the actin cytoskeleton are closely related to the triggering of the plant defense response.

## 1. Introduction

Cotton (*Gossypium* spp.) is one of the most important crops cultivated worldwide. The soil-borne pathogen *Verticillium dahliae* (*V. dahliae*) seriously threatens cotton production. It may cause a severe vascular disease, Verticillium wilt, and results in massive losses in plant mass and lint yield [1,2]. Increasing the plant resistance to Verticillium wilt is a critical challenge worldwide.

Verticillium wilt is characterized that the hyphae of *V. dahliae* propagating and is spread in the cotton woody vascular tissues, where fungicides cannot efficiently reach, which may account for the difficulty in controlling the devastating disease [3,4,5]. Besides blockage of the plant xylem vessel, *V. dahliae* could also synthesize toxic materials and secret proteins, which are thought to play critical roles in the pathogenicity of *V. dahliae* [6,7]. The secreted proteins are usually employed by pathogens, targeting the plant immune system and result in the attenuation of the host PTI (PAMP triggered immunity) or ETI (effector-triggered immunity) [7].

Upon perception of the infection of the *V. dahliae*, cotton plants activate the innate immune system. Multiple crucial defense-related proteins are synthesized and activated to participate in plant defense. For example, cotton plants could secrete NRX1 and CRR1 proteins, functioning in the apoplastic compartment to regulate ROS metabolism and antagonize a *V. dahliae* effector VdSSEP1-mediated fungal disease progression [8,9]. GhLYK1 and GhLYK2, two cell membrane LysM-containing proteins, are induced after *V. dahliae* infection. *GhLYK1* and *GhLYK2* silencing compromise cotton plant resistance to *V. dahliae*. GhLYK2 can induce ROS bursts in plants [10]. Several cotton transcription factors, including MYB108, CBP60g, SARD1 and the recently reported MYB6, have been reported to play crucial roles in cotton defense against *V. dahliae* [11,12,13]. In addition, some *V. dahliae*-responsive genes, such as *MLP28*, *MKK2*, and *BAK1*, have been shown to work in cotton defense against Verticillium wilt [14,15,16]. Exploring the defense-related genes in cotton would provide candidate targets for breading Verticillium wilt-resistant plants.

In plants, the actin cytoskeleton plays a central role in regulating intracellular transport, trafficking, and signaling events, including those associated with cell division and development, organelle movement, and vesicle trafficking [17]. Besides the crucial roles in plant cell development, the actin cytoskeleton is also a dynamic platform for sensing and responding to a diverse array of biotic and abiotic stresses. As a function of the plant immune system, accumulating evidence supports the roles of the cytoskeleton in at least two key aspects of the immune response. (1) It functions in the establishment and maintenance of signaling competent microenvironments; (2) it works in cellular trafficking (organelle, defense-related proteins, and small signaling molecules). In addition, the actin cytoskeleton is dynamically assembled in response to the activation of the plant’s immune system. An immediate increase in actin filament abundance is a conserved feature in the plant PTI. Blocking the actin filament density increase leads to enhanced susceptibility of host plants to pathogenic and non-pathogenic bacteria in Arabidopsis [18]. The actin-related proteins, such as the actin depolymerization factor4 (ADF4), the actin motor protein XI (Myosin XI), and the actin capping protein (CP), were shown to regulate the actin organization in the plant defense against bacterial infection [19,20,21]. Though this evidence indicated that the actin cytoskeleton is closely related to the plant’s immune system, the molecular mechanism that actin functions in the defense response, especially in the plant-fungal resistance, are known to be limited.

LIN-11, Isl1, and MEC-3 (LIM)-domain proteins are widely distributed in eukaryotes ranging from yeast (*Saccharomyces cerevisiae*) to humans [22]. Compared with animal genomes that contain multiple LIM genes, plant genomes encode a limited number of LIM genes. Plant LIM proteins have been shown to function in either actin-binding and bundling activity or in DNA binding to regulate gene expression in plant cell wall synthesis [23,24,25]. Tobacco’s WLIM1, WLIM2, and six Arabidopsis LIM proteins are vital to bundle actin filaments [22,25]. They are also key transcriptional factors through the binding of PAL-box DNA motif to regulate the expression of genes involved in phenylpropanoid biosynthesis and cell division [26,27]. Our previous work characterized four LIM genes in upland cotton (*G. hirsutum*), and of which WLIM1a was shown to have dual roles during fiber development. It can both bind the actin filaments and act as a transcription factor in the secondary cell wall biosynthesis stage [23]. In this study, we characterized another LIM gene in cotton. We found that *GhWLIM1C* expression was up regulated in response to *V. dahliae* infection, and GhWLIM1C and its two LIM domains are predominantly localized at the actin cytoskeleton. The silencing of *GhWLIM1C* in cotton may result in the attenuation of the plant defense response. In all, our results provide new evidence that the LIM protein, GhWLIM1C, could regulate cotton’s fungal defense via regulation assembly of the actin cytoskeleton. These broaden our understanding of the crucial roles of LIM proteins in plant cells.

## 2. Results

### 2.1. Expression of Cotton GhWLIM1C in Response to V. dahliae Infection

**Figure 1:**
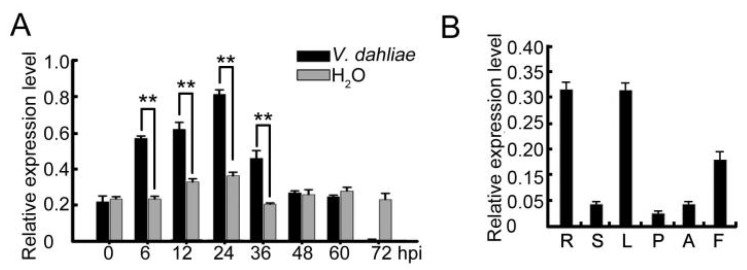
Expression analysis of cotton *GhWLIM1C*. (**A**) qRT-PCR analysis of *GhWLIM1C* expression in cotton roots in response to *V. dahliae* infection. Total RNA was extracted from cotton roots inoculated with V. dahliae spores (1 × 10^6^ conidia/mL) for 0, 6, 12, 24, 36, 48, 60, and 72 h, respectively, and 0 h indicates cotton plants without inoculation with the fungal spores. Error bars represent SD from three independent experiments (*n* = 3). Asterisks indicate statistically significant differences, as determined by Student’s *t*-test (** *p* < 0.01). The experiments were repeated three times with similar results. (**B**) qRT-PCR analysis of *GhWLIM1C* gene expression in root (R), stem (S), leaf (L), petal (P), anther (A), and fiber (F; 12 days post-anthesis) in cotton plants. The expression levels are indicated relative to cotton UBI gene. Error bars represent ± SE of three biological replicates.

Four *LIM* genes were identified in the *G. hirsutum* genome in the previous work [23]. To test whether the expression of these cotton *LIM* genes responds to *V. dahliae* infection, we analyzed their transcript levels in the roots of *G. hirsutum* cotton. Among them, the expression of *GhWLIM1C* (DW492950) was significantly up regulated upon *V. dahliae* infection when compared with H_2_O treatment (Figure 1A). It was up regulated to the highest level at 24 h post-inoculation with the *V. dahliae* spores. As *V. dahliae* is a soil-borne pathogen and the infection was initiated at the cotton root tissue, we then investigated whether *GhWLIM1C* is expressed in the cotton root. The qRT-PCR experiment showed that *GhWLIM1C* was expressed at a higher level in cotton root (R), leaf (L), and fiber (F) compared with the stem (S), petal (P), and anther (A) tissues (Figure 1B).

### 2.2. GhWLIM1C Functions in Defense against V. dahliae Infection

**Figure 2:**
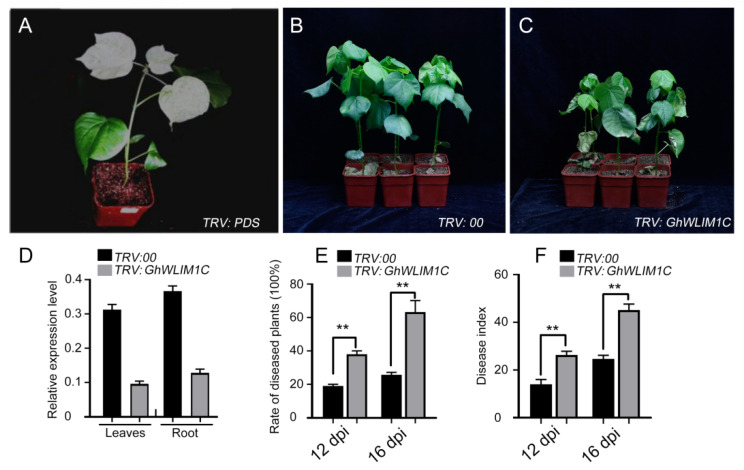
*GhWLIM1C* silencing decreases resistance to *V. dahliae* infection in cotton. (**A**) Preliminary assay of the efficiency of VIGS under our experimental conditions. Ten-day-old cotton plants were infiltrated with *Agrobacterium* carrying *TRV:PDS*. The photographs were taken 2 weeks after infiltration. (**B**,**C**) Phenotypes of control (**B**) and *GhWLIM1C* silenced cotton plants (**C**). Cotton plants were infiltrated with *Agrobacterium* carrying VIGS-control vector (*TRV:00*) or (*TRV:GhWLIM1C*) and inoculated with a suspension of *V. dahliae* spores. Photographs were taken 2 weeks after *V. dahliae* infection. (**D**) qRT-PCR analysis of *GhWLIM1C* expression in cotton leaves and the root tissues infiltrated with VIGS-control vector (*TRV:00*) and *TRV:GhWLIM1C*. Error bars indicate SD from three technical replicates of one biological experiment. The experiments were repeated three times with similar results. (**E**,**F**) Rate of diseased plants and disease index of *TRV:00* and *TRV:GhWLIM1C* cotton plants. Error bars represent the SD of three biological replicates (*n* = 36), and asterisks indicate statistically significant differences, as determined by Student’s *t*-test (** *p* < 0.01).

Considering the expression of *GhWLIM1C* responses to *V. dahliae* infection, we speculate that it may have a function in fungal disease defense. A virus-induced gene silencing (VIGS) system was employed to investigate the function of GhWLIM1C in defense against *V. dahliae* infection in *G. hirsutum* cotton. We preliminary silenced the expression of the cotton phytoene desaturase gene (*PDS*), which is used as a visible marker to monitor the efficiency of VIGS [28]. As shown in Figure 2A, the new growth of true leaves of plants infiltrated with Agrobacterium carrying PDS exhibited an albino phenotype, indicating that the VIGS system worked efficiently. Next, we then inhibited the expression of *GhWLIM1C* in *G. hirsutum*. The qRT-PCR analyses showed that the expression of *GhWLIM1C* was significantly reduced in the leaves and root tissues in the VIGS plants (Figure 2D). *V. dahliae* spores were used to infect both the control (TRV:00) and the *GhWLIM1C* silencing (*TRV:GhWLIM1C*) plants. Phenotypic analysis indicated that down regulation of *GhWLIM1C* expression resulted in reduced resistance to the fungal pathogen (Figure 2B,C). The rate of diseased plants and the disease index of *GhWLIM1C*-silenced plants clearly increased compared with the non-silenced plants (Figure 2E,F).

### 2.3. GhWLIM1C Binds to the Plant Actin Cytoskeleton In Vivo

**Figure 3:**
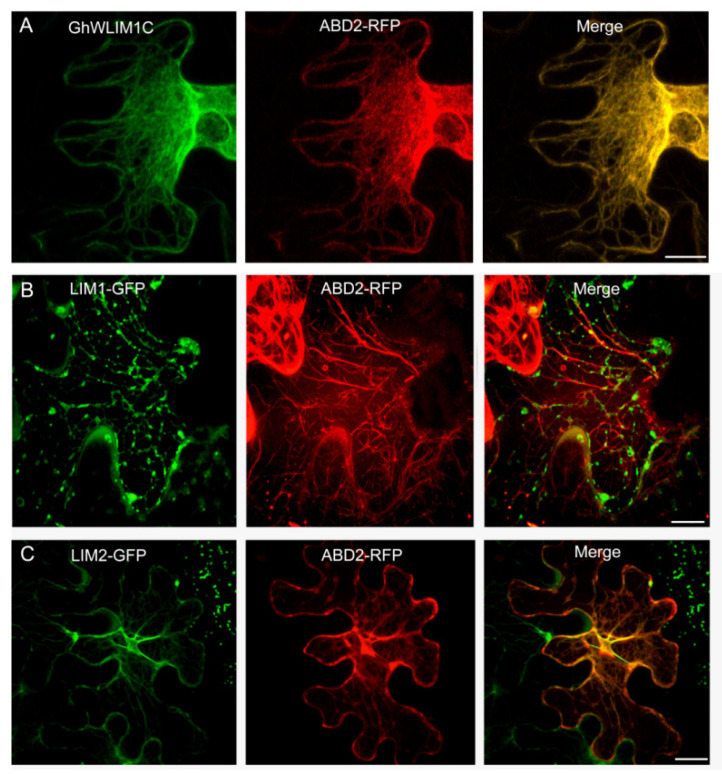
Subcellular localization of GhWLIM1C and its two LIM domains. Colocalization of GhWLIM1C (**A**), LIM1 (**B**), LIM2 (**C**). and ABD2-RFP in the *N. benthamiana* cells. *Agrobacterium* cells containing the indicated pair of GhWLIM1C-GFP, LIM1-GFP, LIM2-GFP, and ABD2-RFP plasmids were coinfiltrated into leaves of *N. benthamiana*. The signal was visualized by confocal microscopy. Bars = 20 µm.

The subcellular localization of GhWLIM1C was investigated in tobacco cells. We transiently expressed GhWLIM1C fused to GFP in *Nicotiana benthamiana* epidermal cells. The fluorescence was observed by confocal laser-scanning microscopy. Fluorescence from the GhWLIM1C-GFP fusion protein was detected as filamentous structures similar to the actin filaments. We then additionally expressed ABD2-RFP as the actin cytoskeleton marker to show the filamentous actin. The results showed that GhWLIM1C-GFP could totally co-localize with the ABD2-RFP-labeled actin cytoskeleton (Figure 3A), indicating that GhWLIM1C may function in the actin organization. Further, we also investigated the subcellular localization of the two LIM domains of GhWLIM1C. We constructed LIM1 and LIM2 domain fusion with GFP and expressed them together with ABD2-RFP in tobacco cells. The results showed that the LIM1 domain GFP was visualized as spot-like structures localized on the actin filaments (Figure 3B), and LIM2-GFP could co-localize with the ABD2-RFP-labeled actin cables (Figure 3C). In all, these results suggested that GhWLIM1C and its two LIM domains could bind to the actin cytoskeleton.

### 2.4. GhWLIM1C Promotes the Formation of Actin Bundles In Vitro

**Figure 4:**
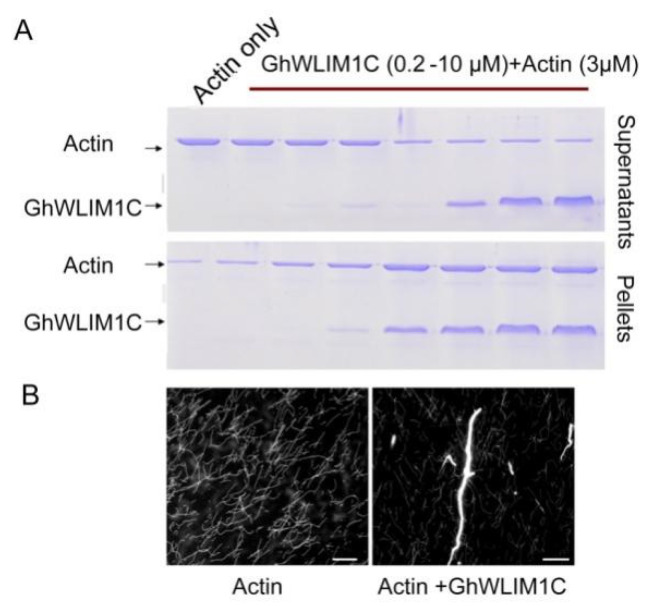
GhWLIM1C bundles actin filaments in vitro. (**A**) Low-speed cosedimentation assay of the actin-bundling activity of GhWLIM1C. The presence of GhWLIM1C in the pellet indicates its cosedimentation with F-actin. Lane 1, actin alone (3 µM); lanes 2 to 8, actin (3 µM) incubated with 0.2, 0.5, 1, 2, 4, 8, and 10 µM GhWLIM1C, respectively. (**B**) Actin bundles visualized by fluorescence microscopy. F-actin alone (**left**); F-actin + 1 µM GhWLIM1C (**right**). Bars = 1 µm.

We performed in vitro experiments to study the biochemical property of GhWLIM1C. We in vitro expressed and purified His-tagged GhWLIM1C from E. coli. A low-speed cosedimentation experiment was conducted to show the property of GhWLIM1C. The rabbit actin was polymerized alone or with an increasing amount of His-GhWLIM1C. After 30 min incubation, the samples were centrifuged at 35,000× *g* for 30 min. Then, the supernatants and pellets were individually separated by SDS-PAGE. The results showed that only a small portion of actin was detected in the pellets. However, it was gradually increased in the pellets along with the elevated amounts of GhWLIM1C (Figure 4A). We then stained the actin samples with the Alexa488-phalloidin and found that the actin assembles as fine filaments (Figure 4B, left). When GhWLIM1C was added, thick actin cables appeared (Figure 4B, right).

**Figure 5:**
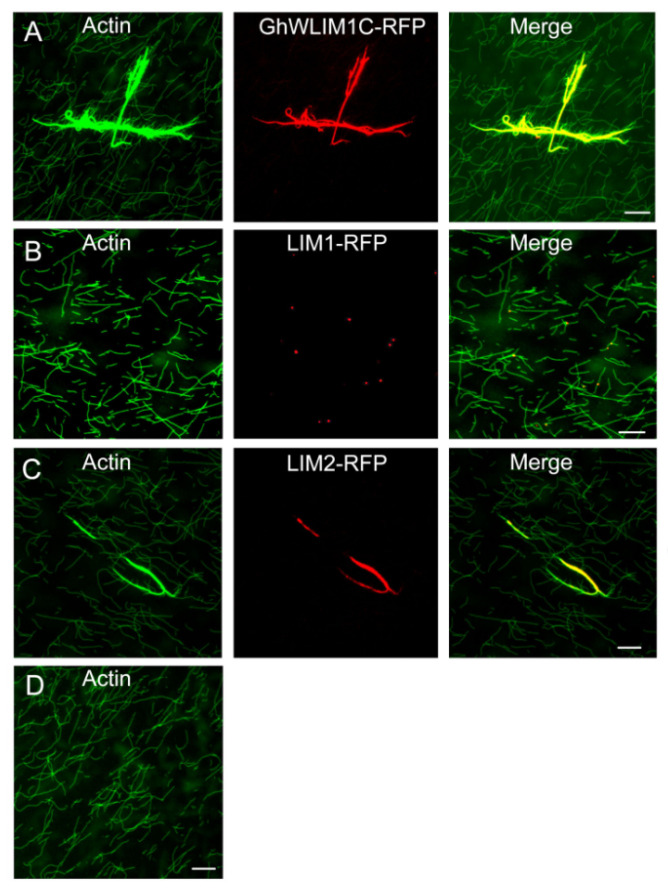
Fluorescence microscopy analysis of F-actin-binding/bundling activities of GhWLIM1C and the two LIM domains. Preassembled F-actin incubated with GhWLIM1C-RFP (**A**), LIM1-RFP (**B**), and LIM2-RFP (**C**). Then, the samples were stained with Alexa488-phalloidin and detected with fluorescence microscopy. Actin alone was used as a negative control (**D**). Bars = 1 µm.

The low-speed cosedimentation experiment shows that GhWLIM1C could bundle the actin cytoskeleton. For further elucidating the mechanism, we in vitro purified His-tagged GhWLIM1C-RFP, LIM1-RFP, and LIM2-RFP. We incubated these proteins with the preassembled actin filaments. Through fluorescence observation, we found that GhWLIM1C formed as robust filamentous structures co-localized with the thick actin bundles (Figure 5A) compared with actin alone forming fine actin filaments (Figure 5D). In addition, we also found that LIM1-RFP was exhibited as spots embedding at the actin filaments (Figure 5B), and LIM2-RFP could form a filamentous structure similar to GhWLIM1C to promote the formation of actin bundles (Figure 5C).

### 2.5. GhWLIM1C May Function Involving in the Activation of Plant Immune Response

**Figure 6:**
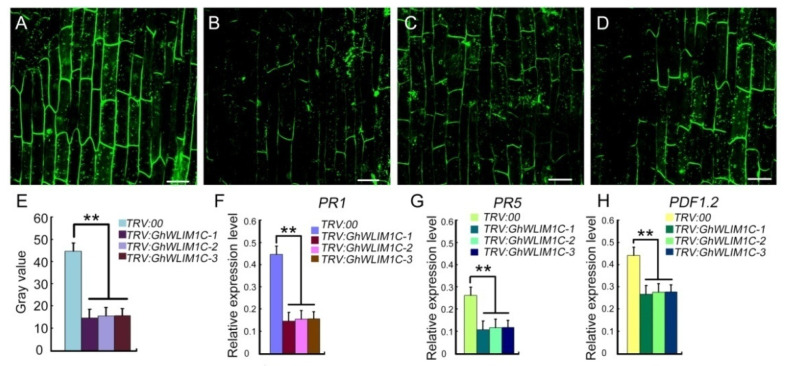
Defense response analysis of *GhWLIM1C*-silencing plants. (**A**–**D**) H2DCFDA staining of the ROS in root cells of the *TRV:00* (**A**) and three *GhWLIM1C*-silencing (**B**–**D**) cotton plants at 72 hpi. Fluorescence of H2DCFDA was detected with confocal microscopy. Bars = 20 µm. (**E**) Quantification of the fluorescence in (**A**–**D**). (**F**–**H**) qRT-PCR analysis of the expressions of defense-related genes, *PR1* (**F**), *PR5* (**G**) and *PDF1.2* (**H**) in the root tissues of the *TRV:00* and three *GhWLIM1C* silencing cotton plants at 72 hpi. Error bars represent the SD of three biological replicates (*n* = 36), and asterisks indicate statistically significant differences, as determined by Student’s *t*-test (** *p* < 0.01).

We next explored the mechanism that GhWLIM1C functions in the plant immune response. The experiments above indicated that down regulation of GhWLIM1C in cotton leads to decreased resistance to *V. dahliae* infection. We then investigated the immune response in the *GhWLIM1C*-silencing plants. We first analyzed the ROS accumulation in control and the *GhWLIM1C*-silencing plants. Through H2DCFDA staining and fluorescence observation, we found that at 72 hpi, in the root cells of the *GhWLIM1C*-silencing plants, less ROS was accumulated compared to the control (Figure 6A–E). Furthermore, we analyzed the expression of several defense-related genes, such as *PR1*, *PR5*, and *PDF1.,2* in the root tissues of the *GhWLIM1C*-silencing plants. The results show that the expressions of these genes were significantly down regulated in the *GhWLIM1C*-silencing plants than in the control (Figure 6F–H).

## 3. Discussion

Cotton Verticillium wilt is a severe vascular disease that may cause a serious cotton loss in both production and quality. Exploring the key factors involved in the defense against *V. dahliae* invasion in cotton can provide candidate genes for generating wilt-resistant *G. hirsutum* cultivars through molecular breeding [29,30]. In this study, we identified a new cotton protein that functions in the plant defense against the fungal pathogen *V. dahliae* and found that cotton GhWLIM1C protein may positively regulate the fungal defense response.

In eukaryotic cells, the actin-binding proteins (ABPs) are indicated to involve in the actin polymerization, dynamics turnover of actin subunits, organization of the actin filaments to network and bundles, in precisely modulating the dynamic actin cytoskeleton [31]. The LIM domain is a conserved domain found in a variety of different proteins. In animals, LIM proteins differ in their functions due to the additional protein domains and are grouped as LIM-hd, LMO, LIM-kinase, and other LIM proteins [32]. Members of LMO and LIM-hd proteins are usually localized in the cell nucleus to act as transcriptional factors in regulating developmental gene expression [33], while some other LIM proteins (other LIM, CRP1) are indicated in the regulation of F-actin organization [34]. In plants, the LIM protein in tobacco NtWLIM1, cotton WLIM1a, and Arabidopsis’ six LIM proteins was localized at the actin cytoskeleton and in the nucleus [23,27,35]. In addition to binding directly to actin filaments for stabilizing the actin cytoskeleton, tobacco NtWLIM1 was found to be a transcriptional regulator that was able to activate the expression of a b-glucuronidase reporter, and the overexpression of *NtWLIM1* led to up regulated expression of PAL-box genes [26]. NtWLIM2 could transcriptionally activate the histone gene, and it shuttles to the nucleus in response to cytoskeletal remodeling [24]. Cotton WLIM1a proteins play dual functions in fiber elongation and secondary cell wall synthesis due to their property of both actin-binding and transcriptional activation of phenolic metabolism [23]. In this study, like the reported plant LIM proteins, GhWLIM1C could also bind to the actin filaments both in vitro and in vivo (Figure 3, Figure 4 and Figure 5). However, in the in vivo experiments, we did not observe an obvious nucleus localization of GhWLIM1C, it seems that it only localized to the actin cytoskeleton (Figure 3A). Considering that GhWLIM1C could both directly bind and bundle the actin filaments in vitro (Figure 4 and Figure 5), we speculate that GhWLIM1C mainly functions in actin remodeling other than gene regulation. A typical plant LIM protein contains two LIM domains, and the two domains were shown to occupy different actin affinities. For example, LIM1 of cotton WLIM1a protein contributed primarily to the actin filament-bundling activity, whereas LIM2 contributed to the DNA-binding activity of the protein [36]. Differently from our experiment, it seems that both LIM1 and LIM2 of GhWLIM1C could bind to the actin filaments (Figure 3B,C; Figure 5B,C); while LIM1 only possesses actin-binding activity (Figure 5B), LIM2 could directly bind and bundle actin filaments (Figure 5C). This indicates that LIM1 and LIM2 may have individual functions though they both contain the same LIM motif.

Emerging evidence showing that the actin cytoskeleton may function crucially in the plant defense response. First, the dynamic rearrangements of the actin cytoskeleton, including the increasing number of actin filamentous and bundling extent elicited after pathogen perception, is a component of plant PTI. Actin filament density and thick actin bundles increase following microbe-associated molecular patterns (MAMP) elf26 treatment, and these dynamic actin changes require pattern recognition receptors sensing the MAMP to trigger the downstream defense response [19]. Secondly, actin could also regulate the strength of the plant’s immunity. The actin cytoskeleton could establish and maintain a signaling-competent microenvironment and traffic plant defense-related organelles, proteins, and small molecules [37]. In our study, we found that GhWLIM1C functions as a pure actin-binding and bundling protein in vivo and in vitro (Figure 3, Figure 4 and Figure 5), and the silencing of this gene leads to decreased resistance to cotton fungal diseases (Figure 2). Thus, we speculate that GhWLIM1C may have a function in the organization of the actin cytoskeleton during plant defense, and its aberrant organization may decrease the downstream immune responses; the generation of ROS in plants cell was reduced, and the expressions of the defense-related genes were weakened (Figure 6). Thus the plant resistances were decreased. Detailed mechanisms about the linking of GhWLIM1C-mediated cytoskeletal rearrangements and plant defense needs further studies. In all, our findings characterized a new actin-related protein, GhWLIM1C, in cotton and provide evidence that GhWLIM1C could regulate plant immune response through regulating actin polymerization.

## 4. Materials and Methods

### 4.1. Cotton Growth and V. dahliae Culture and Infection of Cotton Seedlings

Upland cotton seeds TM-1 (*Gossypium hirsutum*) were grown hydroponically as previously described [38] or cultured in a controlled environment chamber under a 16/8 h photoperiod and at 80% relative humidity. The *V. dahliae* isolate V991 was used in this study [8]. The fungal strain was grown on potato dextrose agar medium. For suspension spore production, mycelia were cultured in liquid Czapek’s medium at 150 rpm/min, 28 °C for 3–5 days. For cotton inoculation, a spore suspension (1 × 10^6^) conidia/mL with deionized water was used. Then, the suspension was injected into the stem of a cotton seedling (10 days old). Root tissues were harvested at 0, 6, 12, 24, 36, 48, 60, and 72 hpi, frozen in liquid nitrogen, and stored at −80 °C until use.

### 4.2. RNA Extraction and RT-qPCR Analysis

Total RNA of cotton leaves and root tissues was extracted using a Plant Total RNA Purification Kit (TransGen Biotech) according to the manufacturer’s protocol. The quantitative reverse-transcription PCR assays were performed using SYBR Green Real-Time PCR Master Mix (Toyobo, Japan), with cotton *UBI* gene as the internal control. All reactions were conducted in triplicate, and primers used in this study are provided in Table S1.

### 4.3. Virus-Induced Gene Silencing

For silencing of *GhWLIM1C* in cotton, we employed the virus-induced gene silencing (VIGS) method as described previously [9]. The gene-specific fragments for *GhWLIM1C* were inserted into pTRV2. *Agrobacterium* cultures harboring pTRV1 and pTRV2- GhWLIM1C were mixed at a 1:1 ratio and injected into 10-d-old cotton cotyledon.

### 4.4. Plasmid Construction and Protein Expression

To construct plasmids harboring 35S-GhWLIM1C-GFP, 35S-LIM1-GFP, and 35S-LIM2-GFP, the corresponding cDNA was amplified using gene-specific primers and cloned into the plant expression vectors pCambia1300-GFP, respectively. All constructs were confirmed by sequencing and then transformed into *A. tumefaciens* strain GV3101. The cDNA fragment contains *GhWLIM1C*, *LIM1*, and *LIM2* were cloned into the bacterial expression vector PET-28a (Novagen/Merck). The recombinant proteins were expressed in *Escherichia coli* strain BL21 (DE3). His-tagged LIM proteins were purified using nickel-nitrilotriacetic acid resin following procedures described by the manufacturer (Qiagen). His-tagged GhWLIM1C, LIM1, and LIM2-RFP recombinant proteins were expressed in *E. coli* and purified following similar procedures as described above. All primers and enzyme sites used in plasmid construction are described in Table S1.

### 4.5. Low-Speed Cosedimentation Assays

Low-speed cosedimentation assays were conducted as described previously [23]. Rabbit muscle actin (3 µM) was incubated at 22 °C for 60 min either alone or with 3 µM GhWLIM1C in KMEI buffer (10× stock: 500 mM KCl, 10 mM MgCl_2_, 10 mM EGTA, and 100 mM imidazole, pH 7.0). For the low-speed cosedimentation assay, the samples were centrifuged at 13,500× *g* for 30 min at 4 °C. The proteins in the supernatants and pellets were separated by SDS-PAGE and visualized with Coomassie Brilliant Blue R250.

### 4.6. In Vitro Assembly and Observation of the Actin Cytoskeleton

Rabbit muscle actin (3 µM) was incubated at 22 °C for 60 min either alone or with 3 µM GhWLIM1C in KMEI buffer (10× stock: 500 mM KCl, 10 mM MgCl_2_, 10 mM EGTA, and 100 mM imidazole, pH 7.0). Then, the polymerized F-actin was incubated with LIM proteins (GhWLIM1C, GhWLIM1C-RFP, LIM1-RFP, and LIM2-RFP) for 30 min. After that, these samples were stained with 0.066 µM Alexa488-phalloidin (Molecular Probes) for 10 min. Fluorescence signals were observed under confocal microbiology (Zeiss 880).

### 4.7. Fluorescent Staining and Confocal Lasers Canning Microscopy

For observation of LIM subcellular localization of GhWLIM1C, LIM1, and LIM2, the GFP-tagged LIM proteins were expressed in *N. benthamiana* leaves through *A. tumefaciens* strain GV3101 injection. Then the fluorescent signals were visualized under a confocal microscope after two days of expression.

## Data Availability

Not applicable.

## Supplementary Materials

The following supporting information can be downloaded at: https://www.mdpi.com/article/10.3390/plants11141828/s1, Table S1: Primers used in the study.
