# Supplementary material for: Silencing of a Cotton Actin-Binding Protein GhWLIM1C Decreases Resistance against Verticillium dahliae Infection
Source: Plants (Basel). 2022 Jul 12;11(14):1828. doi: 10.3390/plants11141828 (PMC9316592; doi:10.3390/plants11141828)
Supplement: Supplementary file 1 [file plants-11-01828-s001.zip › plants-1803569-supplementary.pdf]

plants MDPI

Table S1. The nucleotides highlighted in red indicate the enzyme site for construction.

**For localization**	
1300-GhWLIM1C-GFP-F	CGACGGCCAGTGCCAAGCTTATGGCATTTGCAGG
1300-GhWLIM1C-GFP-R	GCCCTTGCTCACCATCCCGGGTTAAGATTCAGCAGCA
1300-LIM1-GFP-F	CGACGGCCAGTGCCAAGCTTATGGCATTTGCAGG
1300-LIM1-GFP-R	GCCCTTGCTCACCATCCCGGGTCTCAAGACTACCAGTTCTT
1300-LIM2-GFP-F	CGACGGCCAGTGCCAAGCTTATGGGCATGTTTGGTG
1300-LIM2-GFP-R	GCCCTTGCTCACCATCCCGGGTTAAGATTCAGCAGCA
**For protein expression**	
pET-28a-GhWLIM1C-F	GACAGCAAATGGGTCGCGGATCCATGGCATTTGCAGG
pET-28a -GhWLIM1C-R	GCTCGAGTGCGGCCGCAAGCTTTTAAGATTCAGCAGCA
pET-28a-GhWLIM1C-RFP-F	GACAGCAAATGGGTCGCGGATCCATGCAGTCCGGAATCTCAGG
pET-28a-GhWLIM1C-RFP-R	GCTCGAGTGCGGCCGCAAGCTTGCTCCCAACACTGTCT
pET-28a-LIM1-RFP-F	GACAGCAAATGGGTCGCGGATCCATGGCATTTGCAGG
pET-28a-LIM1-RFP-R	GCTCGAGTGCGGCCGCAAGCTTTCTCAAGACTACCAGTTCTT
pET-28a-LIM2-RFP-F	GACAGCAAATGGGTCGCGGATCCATGGGCATGTTTGGTG
pET-28a-LIM2-RFP-R	GCTCGAGTGCGGCCGCAAGCTTTTAAGATTCAGCAGCA
For qRT-PCR	
WLIM1C-F	AATCTAAATCACTCCATTTC
WLIM1C-R	GTAGCTATTGGTTTCTCA
UBI-F	CACCATTGATGACAGAGATTTTA
UBI-R	CTGAATCTTCGCTTTCACGTTATC
PR1-F	AATTGGTCCTTCCCTCCCATG
PR1-R	AACCTGCTGCGCACCTGTTAGAGTTG-3
PR5-F	AGCCGCCTCA GCGTTTATTT TTAAA
PR5-R	TACGAGCCATGGCTGTGCCAGCAG
PDF1.2-F	ATTCCTCGAAAAATAATAATCTAG
PDF1.2-R	TAAATATTATTATAACCGAGCGAA
